# Successful laparoscopic cholecystectomy for gallbladder hemorrhage from a Dieulafoy lesion in a patient on hemodialysis: a case report

**DOI:** 10.1186/s40792-023-01628-5

**Published:** 2023-03-24

**Authors:** Yuu-ichi Yano, Tomohiro Iguchi, Shota Sato, Norifumi Iseda, Shun Sasaki, Yasuhiro Abe, Tomohiro Nakayama, Takuya Honboh, Seiya Kato, Noriaki Sadanaga, Hiroshi Matsuura

**Affiliations:** 1 Department of Surgery, Saiseikai Fukuoka General Hospital, 1-3-46 Tenjin, Chuo-Ku, Fukuoka, 810-0001 Japan; 2 Department of Internal Medicine, Saiseikai Fukuoka General Hospital, 1-3-46 Tenjin, Chuo-Ku, Fukuoka, 810-0001 Japan; 3 Department of Radiology, Saiseikai Fukuoka General Hospital, 1-3-46 Tenjin, Chuo-Ku, Fukuoka, 810-0001 Japan; 4 Division of Pathology, Saiseikai Fukuoka General Hospital, 1-3-46 Tenjin Chuo-Ku, Fukuoka, Japan

**Keywords:** Gallbladder, Hemorrhage, Dieulafoy lesion, Hemodialysis, Laparoscopic cholecystectomy

## Abstract

**Background:**

Patients on long-term dialysis are prone to hemorrhagic complications, particularly uremic bleeding, but gallbladder hemorrhage is rare, even in patients on dialysis. There have been occasional reports of a Dieulafoy lesion being a cause of gastrointestinal hemorrhage, but its occurrence within the gallbladder is quite rare. This report describes a case of gallbladder hemorrhage from a Dieulafoy lesion in a patient on hemodialysis that was diagnosed early and successfully treated by laparoscopic cholecystectomy.

**Case presentation:**

The patient was a 68-year-old woman on long-term hemodialysis with end-stage renal failure who presented with epigastralgia and back pain. There was no history of trauma or oral administration of antiplatelet or anticoagulant agents. There were no signs of an inflammatory reaction or hyperbilirubinemia. Contrast-enhanced computed tomography revealed a slightly hyperdense area in the distended gallbladder and extravasation within the gallbladder lumen but no gallstones. A severe atherosclerotic lesion was also found. She was diagnosed to have gallbladder hemorrhage and emergency laparoscopic cholecystectomy was performed. Although the postoperative course was complicated by drug fever, she was discharged on postoperative day 10 in a satisfactory condition. Histology revealed hemorrhagic ulceration with an exposed blood vessel accompanied by abnormal arteries in the submucosa. Arteriosclerosis with eccentric intimal hyperplasia in a small-sized artery was also seen. The diagnosis was gallbladder hemorrhage from a Dieulafoy lesion.

**Conclusions:**

A Dieulafoy lesion should be kept in mind as a cause of gallbladder hemorrhage in a patient with severe arteriosclerosis and a bleeding diathesis, particularly if on dialysis, and treated as early as possible.

## Background

Gastrointestinal hemorrhage has been described in patients on hemodialysis in association with peptic ulcer (20–30%), gastritis (20%), and telangiectasia of the stomach, duodenum, jejunum, and colon (20–30%) [1]. However, gallbladder hemorrhage is rarely encountered even in patients on dialysis with bleeding diathesis. The most common causes of gallbladder hemorrhage are cholecystitis, gallstones, trauma, tumor, pseudoaneurysm, and use of antiplatelet or anticoagulant agents [2], and there have been reports of gallbladder hemorrhage as a fatal complication of cholecystitis or gallstones in patients on hemodialysis [3, 4].

Another known cause of gastrointestinal hemorrhage is a Dieulafoy lesion, which is an abnormally dilated small artery that runs a tortuous course within the submucosa and accounts for 1–2% of all gastrointestinal hemorrhages [5]. This lesion is usually located in the stomach and is extremely rare in the gallbladder. In this report, we describe a case of gallbladder hemorrhage from a Dieulafoy lesion in a patient on hemodialysis who was diagnosed early and treated successfully by laparoscopic cholecystectomy.

## Case presentation

**Fig. 1:**
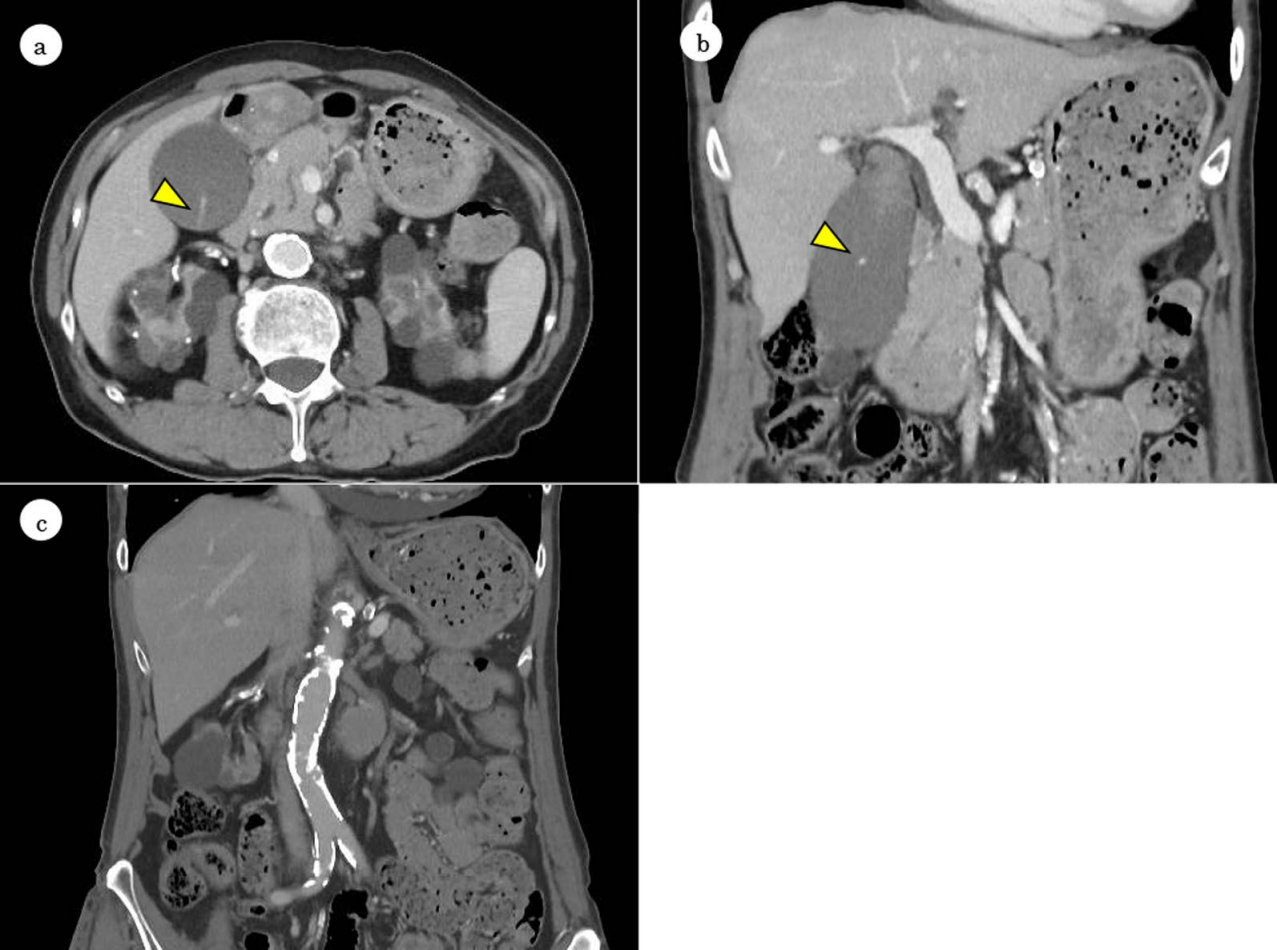
Contrast-enhanced computed tomography scans showing slightly hyperdense material and active extravasation (arrowhead) within a distended gallbladder lumen. There is no evidence of aneurysm of the cystic artery, gallstones, or ascites (**a**, **b**). Severe calcification is present in the aorta and the peripheral arteries (**c**)

A 68-year-old woman presented with dizziness and diagnosed with end-stage renal failure caused by nephrosclerosis and aplastic anemia 23 years ago. She had been on hemodialysis and treated with anabolic steroids and prednisolone for 22 years. While on hemodialysis and treatment with heparin at our hospital, she developed epigastralgia and back pain. There was no history of trauma or oral antiplatelet or anticoagulant therapy. Physical examination revealed a body temperature of 36.8 °C, a pulse of 66 beats/min, a blood pressure of 190/86 mmHg, a respiratory rate of 20 breaths/min, and oxygen saturation of 97% on room air. There were no electrocardiographic changes. She had localized pressure pain in the right hypochondrium and Murphy’s sign was positive. Laboratory data showed elevated alkaline phosphatase (139 U/L) and gamma-glutamyl transpeptidase (173 U/L) but no inflammatory reaction or hyperbilirubinemia. Contrast-enhanced computed tomography (CT) revealed a distended gallbladder and slightly hyperdense material and extravasation within the gallbladder lumen (Fig. 1a, b) but no evidence of cystic artery aneurysm. The extrahepatic bile duct was not dilated and there was no evidence of gallstones or ascites. A severely atherosclerotic lesion was also found (Fig. 1c). These imaging findings were compatible with gallbladder hemorrhage or hemorrhagic cholecystitis.

**Fig. 2:**
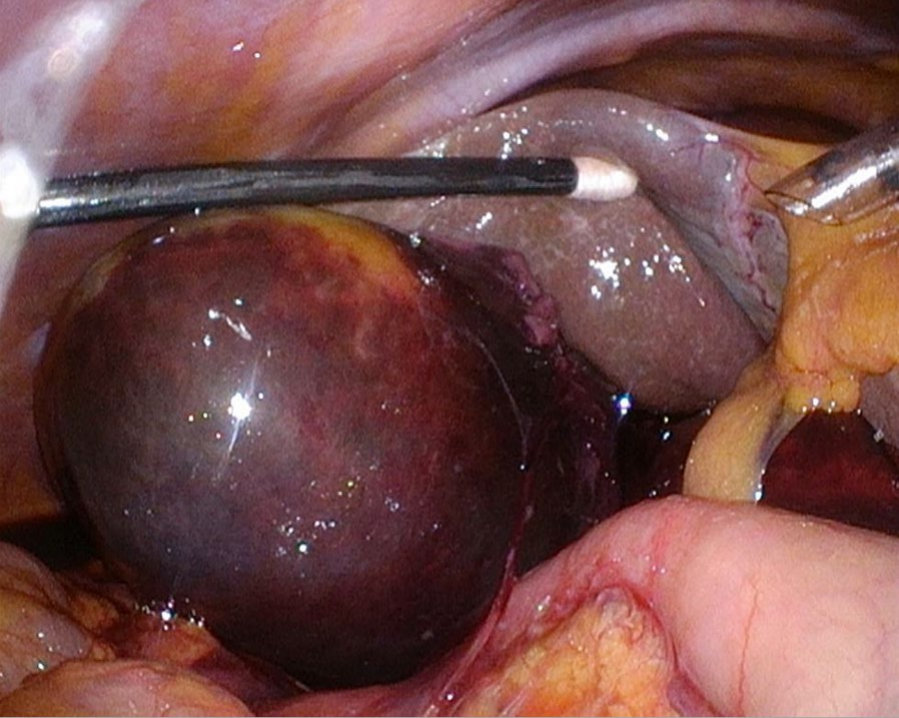
An intraoperative photograph showing a tense gallbladder with a laceration of the serosa accompanied by hemorrhagic ascites

Considering the active bleeding and the patient's good general condition, we elected to perform emergency laparoscopic cholecystectomy, which was carried out using four ports. Intraoperative findings revealed a tense gallbladder, a small amount of hemorrhagic ascites, and a laceration in the gallbladder serosa (Fig. 2). The gallbladder was too tense and difficult to grasp, so a small hole was made in the gallbladder fundus and dark red-colored fluid was aspirated. The cystic duct and cystic artery were identified and carefully divided, and the cholecystectomy procedure was completed. The operation time was 92 min. The postoperative course was complicated by drug fever, with meropenem as the suspected culprit agent, which resolved after a switch to ampicillin and sulbactam. The patient was discharged from hospital on postoperative day 10.

**Fig. 3:**
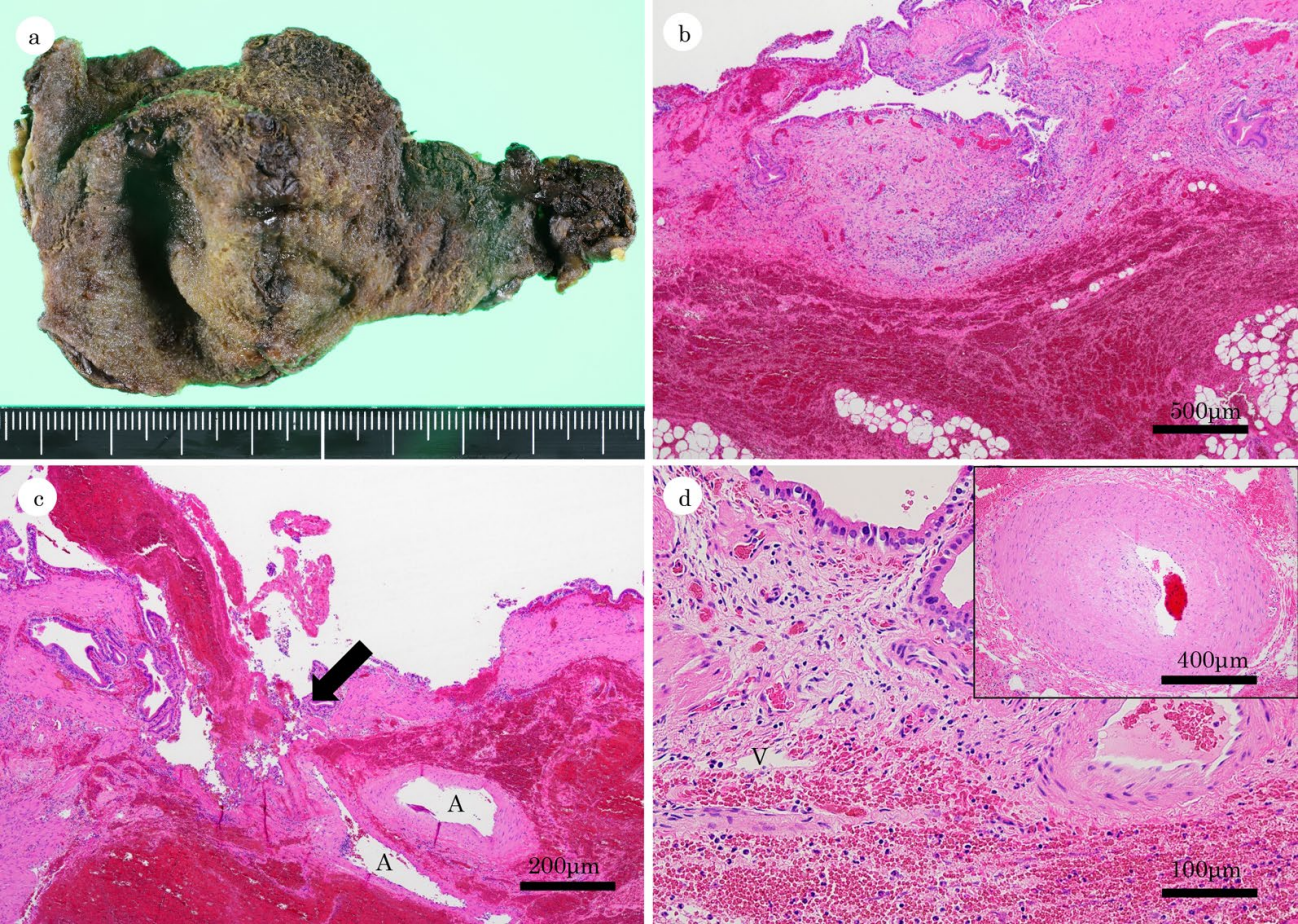
Macroscopic and microscopic findings. Macroscopic view of the luminal surface of the removed gallbladder showing extensive hemorrhage attached on the eroded mucosal tissue (**a**). Histology (low power view) shows widespread hemorrhage in the subserosa (**b**). Histology (medium power view) shows hemorrhagic ulceration with an exposed blood vessel (arrow). Abnormal distribution of arteries (A) was noted in the submucosa (**c**). Histology (high power view) showing hemorrhagic extravasation from a small vein (V). Arteriosclerosis with eccentric intimal hyperplasia of a small-sized artery was also seen in the subserosa (inset) (**d**)

Macroscopic examination revealed extensive hemorrhage that was attached to the eroded mucosa of the gallbladder (Fig. 3a). Histology showed marked hemorrhage characterized by red blood cells that were widely spread in the subserosal layer (Fig. 3b) and hemorrhagic ulceration with an exposed blood vessel accompanied by abnormal arteries in the submucosa (Fig. 3c). Hemorrhagic extravasation from a small vessel, suggesting bleeding diathesis, and arteriosclerosis with eccentric intimal hyperplasia of a small-sized artery in the submucosa were also seen. There was a mild chronic inflammation (Fig. 3d). The diagnosis was gallbladder hemorrhage from a Dieulafoy lesion.

## Discussion

Gallbladder hemorrhage is a rare entity and is most commonly associated with gallstones, acute cholecystitis, and use of antiplatelet or anticoagulants [6–8]. In this case, there was no obvious evidence of gallstones or neoplastic lesions, oral anticoagulant medication, or episodes of trauma. Although the patient had been on long-term hemodialysis, there was no laparoscopic or histological evidence of acute inflammation of the gallbladder. In general, patients on long-term dialysis are prone to bleeding complications; uremic hemorrhage is a typical clinical complication and is associated with uremic toxins, anemia, platelet dysfunction, comorbidities, use of antiplatelet or anticoagulant medications, and aging [3, 9, 10]. However, gallbladder hemorrhage is uncommon.

A Dieulafoy lesion is a tortuous arteriole in the submucosa that bleeds as a result of mucosal erosion in an absence of inflammation and is most often found in the stomach [11]. Uremic bleeding may also be a risk factor for a Dieulafoy lesion but there are few reports of this lesion being found in the stomach in patients on dialysis [12–14]. Furthermore, a Dieulafoy lesion occurring within the gallbladder has rarely been reported in the literature, with only eight previously published cases [15–17], and ours is the first case of a Dieulafoy lesion in the gallbladder in a patient on hemodialysis.

Although it has been suggested that a Dieulafoy lesion is caused by vascular dysplasia resulting from chronic inflammation, which leads to thrombosis and necrosis of the arterial wall [18], the etiology of Dieulafoy lesion is still unknown. Mucosal erosion is associated with ischemic injury that is possibly related to cardiovascular disease and further weakens a fragile point in the wall of the gastrointestinal tract [19]. In this case, CT revealed severe arteriosclerosis, and histology showed some arterial branches with eccentric intimal hyperplasia in the submucosa. Patients on long-term hemodialysis often have advanced arteriosclerosis, which is caused by calcification as a result of abnormal phosphate and calcium metabolism [20]. It is possible that the increase in abnormal arterial branches in the submucosa in this case resulted from chronic ischemia of the gallbladder wall caused by arteriosclerosis associated with long-term hemodialysis, but this would not explain why the arterial wall was disrupted.

Gallbladder hemorrhage is difficult to diagnose on the basis of clinical findings alone because its symptoms mimic those of other common hepatobiliary diseases [3]. However, it is associated with high morbidity and mortality rates because of gallbladder perforation and massive hemorrhage [17]. Early diagnosis is important to facilitate urgent surgical management. CT plays a crucial role in the diagnosis of gallbladder hemorrhage and may show high-density fluid within the gallbladder lumen [21]; however, it can be difficult to diagnose because it looks similar to gallbladder tumor or sludge. Magnetic resonance imaging can distinguish blood from gallstones and sludge [22], but there is no grace period in such cases. Pandya and O’Malley demonstrated that early phase contrast-enhanced CT could help to detect active hemorrhage within the lumen of the gallbladder [23]. Nevertheless, the cause of the disease is often not considered for diagnosis. A Dieulafoy lesion should be considered in the differential diagnosis in patients with risk factors, such as hemodialysis.

Emergency surgical intervention for gallbladder hemorrhage can be an effective option to stop bleeding and gallbladder perforation. Recently, laparoscopic cholecystectomy has been reported as the surgical intervention for gallbladder hemorrhage [7]. In the present case, since the patient was diagnosed early and the general condition was relatively good, emergency laparoscopic cholecystectomy was performed. However, in high surgical risk patients, conservative treatment can also be considered. When the patient's condition requires emergency resuscitation and surgery is not feasible, successful transcatheter embolization may achieve hemostasis [24]. Cholecystostomy is also an option for patients who are not surgical candidates [25], while it is to be noted that cholecystostomy may be less successful than cholecystectomy because of inadequate drainage [23]. Conservative treatment is often unsuccessful in cases of gallbladder hemorrhage from a Dieulafoy lesion [26]. Therefore, after hemodynamic stabilization, the indication for cholecystectomy is considered necessary.

## Conclusions

We have encountered a case of gallbladder hemorrhage from a Dieulafoy lesion in a 68-year-old patient on hemodialysis who was treated successfully by laparoscopic cholecystectomy. In view of the potentially serious complications, a Dieulafoy lesion should be diagnosed and treated as early as possible. This lesion should be kept in mind as a cause of gallbladder hemorrhage in patients with severe arteriosclerosis and bleeding diathesis, particularly those on dialysis.

## Data Availability

The authors declare that all the data in this article are available within the article.

## Abbreviation

CT: Computed tomography
